# The bottom–up approach to defining life: deciphering the functional organization of biological cells via multi-objective representation of biological complexity from molecules to cells

**DOI:** 10.3389/fphys.2013.00369

**Published:** 2013-12-18

**Authors:** Sathish Periyasamy, Alex Gray, Peter Kille

**Affiliations:** ^1^Cardiff School of Computer Science and Informatics, Cardiff UniversityCardiff, UK; ^2^Organisms and Environment Division, Cardiff School of Biosciences, Cardiff UniversityCardiff, UK; ^3^Department of Bioinformatics, King Abdullah International Medical Research Center, National Guard Health AffairsRiyadh, Saudi Arabia

**Keywords:** multi-objective, cohesive structures, temporal modeling, cellular physiology, adaptation

## Abstract

*In silico* representation of cellular systems needs to represent the adaptive dynamics of biological cells, recognizing a cell's multi-objective topology formed by temporally cohesive intracellular structures. The design of these models needs to address the hierarchical and concurrent nature of cellular functions and incorporate the ability to self-organize in response to transitions between healthy and pathological phases, and adapt accordingly. The functions of biological systems are constantly progressing, due to the ever changing demands of their environment. Biological systems meet these demands by pursuing objectives, aided by their constituents, giving rise to biological functions. A biological cell is organized into an objective/task hierarchy. These objective hierarchy corresponds to the nested nature of temporally cohesive structures and representing them will facilitate in studying pleiotropy and polygeny by modeling causalities propagating across multiple interconnected intracellular processes. Although biological adaptations occur in physiological, developmental and reproductive timescales, the paper is focused on adaptations that occur within physiological timescales, where the biomolecular activities contributing to functional organization, play a key role in cellular physiology. The paper proposes a multi-scale and multi-objective modeling approach from the bottom–up by representing temporally cohesive structures for multi-tasking of intracellular processes. Further the paper characterizes the properties and constraints that are consequential to the adaptive dynamics in biological cells.

## Introduction

Biomolecules give rise to living entities by self-ordering/organizing into coordinated biochemical activities, whose ultimate outcome is the production of life (Abel, 2011). The multi-dimensional problem that needs to be resolved, involves balancing the myriad of biological activities at various levels of biological organization to result in a viable living system. However, a suitable resolution must exist within the “organizational space” defined by the constraints of each constituent biomolecule and their activities. At the cellular level, solutions to the adaptive requirement emerge from the simultaneous adaptation of multiple and mostly conflicting objectives formed by competition amongst temporally cohesive structures (i.e., functional units). This is subjected to various control mechanisms (forms of feedback and reinforcement mechanisms which facilitate self-organization and selection, respectively), which act as regulators in space and time. These regulators can exchange information directly via feedbacks and indirectly via reinforcement mechanisms. These control mechanisms have a perception at their system level, that one outcome is qualitatively better than another at this level, but cannot determine whether this will be true at higher levels and thus cannot determine, if it will lead to an absolute fitness specific to the requirements. Moreover, these mechanisms don't inherently know what is the optimum solution, or even if one exist (Eberhart and Shi, 2007). Although each of the objectives will not have an optimal solution, the solutions observed will ultimately satisfy the requirements in a sustained biological equilibrium. However, challenges to this equilibrium, which exceed the capacity of a specific system to compensate, will create a pathological process, resulting in the multi-objective re-organization manifested as biological adaptation. Further, pathological processes have become an integral part of biological adaptation due to failure in achieving objectives caused by unanticipated constraints. Moreover there will be multiple biological solutions, which represent different “trade-offs” among the objectives and constraints, associated with the biological system. The preferred solution will vary depending on changing requirements (i.e., criteria) exerted by the biological system's dynamic environment. Biological cells are mostly driven toward meeting the objectives which are temporal in nature and strive to explore and exploit solutions with respect to time within the bounds of the spatial constraints.

Biological adaptations occur within physiological, developmental and reproductive timescales. Although this research is focused on physiological timescales, it is useful to understand how biological systems are organized to adapt across these timescales, i.e., how information regarding performance between biological systems and the environment are exchanged across these scales. Biological systems dynamically adapt to multiple objectives concurrently. This is a process facilitated by their constituents forming spatial and temporal cohesive structures. The objectives and cohesive structures of biological systems are constantly adapting due to the ever changing demands of their environment. These objectives are imposed by the environment, which consists of physical, chemical and biological elements of the individual biological systems. Biological systems are driven toward meeting the imposed objectives aided by their constituents, giving rise to biological processes which are perceived as biological functions. Biological tasks emerge through the formation and development of spatially and temporally cohesive structures when pursuing these objectives. Due to the nested nature of cohesive structures, the biochemical tasks appear to be concurrent and mutually dependent, which leads to the manifestation of pleiotropy and polygeny in cells. Pleiotropy is defined as one gene giving rise to two or more unrelated phenotypic traits (Stearns, 2010) and polygeny is defined as a phenotypic trait controlled by more than one gene. These objectives, on which the selective pressure is imposed, are eventually organized into a spatially and temporally cohesive hierarchy forming the biological organization strata, where the amount of time required in pursuing the objectives increases, when moving up the hierarchy (Schnell et al., 2007; Noble, 2008; Dada and Mendes, 2011). The cellular activities are hierarchically organized into various basic tasks, which merge to form the complex and greater tasks of the cell, thus reflecting the nested nature of intracellular cohesive structures. Hence, managing competition and cooperation of these structures will require coordination via hierarchical regulation (Westerhoff and Palsson, 2004) that includes transcriptional, post-transcriptional, translational and post translational regulators.

The paper evaluates the mechanisms of biological adaptation and specifies two categories of goals/objectives, which define these tasks and drive the adaptive process. The aim is to elicit requirements for *in silico* representations of the adaptive dynamics from molecules to cell. An appropriate systems biology approach (Bruggeman and Westerhoff, 2007) will have to be adapted to model the self-organization of biomolecular activities in order to study the emergence of intracellular functional organization. Since it requires a mechanism based explanation, it has to be mechanistically modeled using a bottom–up approach which integrates molecular level information. Modeling at the level of molecular resolution will require representing the molecular properties together with the spatial and temporal constraints of the cellular environment. Since systems biology addresses the missing links between molecules and physiology (Bruggeman and Westerhoff, 2007), it has to integrate experimentation and theoretical frameworks through computational models to study complex biological phenomena. Hence, the paper states how diverse biomolecular activities are groped based on identifying the functionally cohesive structures and measuring the performance of these structures to assess their functions. Further, the concept of Pareto Optimal Frontier is used to assess the level of organization among numerous conflicting biochemical activities.

The paper also evaluates the properties and constraints that are consequential to the adaptive dynamics in biological cells. Further the paper describes the multi-objective nature of biological systems, the constraints involved in pursuing these objectives, and the hierarchical nature of biological systems by simplifying cellular complexity via the construction/deconstruction of basic objectives/tasks into mutually dependent complex global tasks. To model uncertainty, concurrency, self-organization and emergence in intracellular biochemical activities, a suitable modeling formalism will have to be utilized. The collective dynamics approach aided by multi-objective topology, has the ability to represent concurrency and functional hierarchy. The model captures the diverse activities of functional products that occur concurrently in space and time, and avoid the combinatorial explosion inherent in network representations (Takahashi et al., 2005; Felix and Wagner, 2006; Kitano, 2010). Multi-objective topology provides a concurrent and hierarchical view of biological systems, whereas network topology provides a sequential and horizontal view of biological systems.

## The characteristics and properties of biological cell

**Figure 1:**
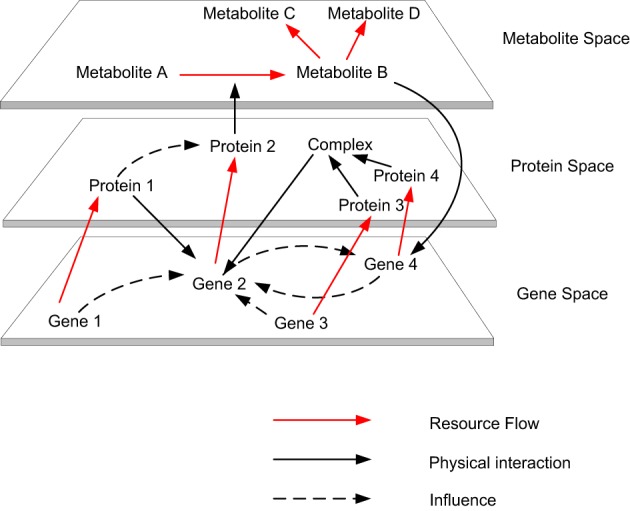
**The autocatalytic cycles traverse across gene space to metabolite space**. Biomolecular activities occurring within the gene, transcript, protein and metabolite space contribute to the organization of a biological cell. These activities form various causalities (i.e., causal links amongst events), which form the organizational closure of the cell.

Modeling and simulating the multi-level dynamics of biological systems are one of the most complex endeavors in systems biology studies, due to the fact that biological processes consist of multi-level spatial and temporal scales (Bassingthwaighte et al., 2006; Schnell et al., 2007; Noble, 2008). Living systems are the most complex systems known in nature, which is due to the multiple levels of constraints associated with them. Living systems are constrained by physical laws, like non-living systems and also have additional levels of constraints associated with complex biological processes (Abel, 2011). These two levels constitute the fundamental and organizational principles, which are required to model the complexity of biological cells from the bottom up (Kitano, 2007). When considering the relationship between individual biomolecules and the cells to which they contribute, we can identify their resemblance to complex, dynamic, self-organizing, adaptive, concurrent, robust, reactive (Efroni et al., 2003) and proactive systems (Michener et al., 2001). Some typical properties of complex systems include dynamism, emergent behavior, non-linearity, multi-stability, nested organization, feedbacks (i.e., horizontal and vertical) and scale freeness (Dubitzky, 2006). Biomolecular activities occurring within the gene, transcript, protein and metabolite space contribute to the organization of a biological cell. These activities form various causalities (i.e., causal links amongst events), which form the organizational closure of the cell (Shapiro, 2007) (see Figure 1). This closure is different from thermodynamic closure, which is observed in isolated systems. Although biological systems are organizationally closed, they are thermodynamically open systems that exist far from thermodynamic equilibrium by exchanging matter and energy with their environment (Bachmair et al., 1986; Van Regenmortel, 2007; Yafremava et al., 2013). For example, at the organizational level various resources (e.g., metabolites) are consumed and produced by various enzyme mediated reactions, and if this is visualized by comparing every resource against every reaction in a matrix, the complex dependencies between enzyme mediated reactions at the thermodynamic level can be observed. As a physical system the laws of thermodynamics direct cellular metabolism (Wolfe, 2001; Alberty, 2010), as a chemical system competition, cooperation and coordination stabilizes cellular metabolism and as living system adaptability, robustness and efficacy ensure persistence of the system.

## Task formation and integration of functional units in cells

Modularization is a way of simplifying complex systems into a set of simple systems using functional abstractions, which constitute the functional units. To this end various criteria for simplifying the complex biochemical activities of life have been proposed, using modularity to encapsulate biological complexity. One such modularity is based on the cellular component and biological processes, which is not compatible in representing the evolution of different functional products and gene families, as functional products are “co-opted” for new tasks beyond what is believed to be their original role (Hodgkin, 1998). Moreover, the long standing question is to what extent the concept of modularity introduced for engineered systems, provides realistic and useful abstractions for systems organized by biological adaptation from physiological to reproductive timescales (Szallasi et al., 2006). Although modularity can be observed in the biological organization strata, in terms of perceivable and spatially cohesive entities (molecules, organelles, cells, organs, and individuals), their applicability in modularizing the intracellular activities of functional products into functional units and cellular processes is doubtful. Intracellular functions that lack spatial boundaries are temporal phenomena, which emerge from the causally linked temporal cohesive structures. A logical approach to simplify cellular processes, is by constructing/deconstructing these processes into objectives/tasks, on which the selective pressure is imposed. Further, the modularity is concealed, due to mutual dependency amongst the higher level tasks. The effects of mutual dependency amongst the objectives/tasks, which occur due to the presence of degenerate and redundant factors, and the convergence and divergence of causal effects of biomolecular activities, adds to the complexity of modularizing biochemical activities. Mutual dependencies complicate the process of identifying the degree of orthogonality (i.e., independence), which facilitates the modularization from molecular resolution to cellular resolution via deconstruction of objectives into basic and molecular tasks required to pursue them. The emergence of global cellular behavior is a result of functional products, which are specialized to pursue their intended tasks. Further, acts of cooperation, competition and coordination emerge from the collective dynamics of functional products. These actions are not mutually exclusive, rather they contribute concurrently to the pursuit of various collective tasks of the cell and higher multi-cellular organizations. The criteria used to modularize the interactions among functional products, are based on performance/fitness interactions, which emerge out of competition and cooperation among functional products. This is the mechanism by which evolution formed and evolved collaborative groups (Axelrod and Hamilton, 1981; Axelrod, 2006; Nowak, 2006) containing one or more species of functional product. These functional products within a group cooperate with each other for a common objective/task. In physiological timescales competitive and cooperative adaptation among various biomolecular species is ubiquitous amongst their activities. While inverse performance/fitness interaction exists between the competing biomolecular species, positive performance/fitness interaction will exist among cooperating biomolecular species. Direct and inverse fitness interactions can reveal the organization of the objective hierarchy in order to construct/deconstruct the tasks between molecular resolution and cellular resolution. Further this relationship is appropriate to model the impact amongst various species of biomolecule's activity on the intracellular and cellular level processes, as a whole.

Temporal networks are formed due to convergence and divergence of causalities. The interaction between a common transcription factor and various *cis* regulatory sites, is an indication of divergence in causality. The presence of divergence points in biochemical networks is an indication of competition for a common substrate and this leads to conflicts among higher level cellular tasks/objectives. Shared resources are a major cause of conflicts in intracellular organization. A basic task or a cooperative module (functional unit) in biochemical activities is defined as a group of one or more species of functional product collaborating for a common objective. These modules will have the characteristic, that every functional product's performance will have a beneficial effect on the other and the whole group's performance. The absence of any one member species of the group will have no value for the existence of the remaining member species of the group (all or nothing phenomena). In molecular complexes the participating biomolecular species form cooperative groups. In the context of metabolic networks, this is a pathway which exists between two junction points. This will be the basic functional unit, from which higher levels of functional units are assembled. The fitness, at the functional product level, is a function of its efficiency and stability. Efficiency depends on the product's affinity for interaction, and the time and energy requirements for its activity. An improved performance for one competing unit implies a decreased performance for another unit. Hence this has an inhibitory effect on other competing functional units. Further, biomolecules are forced to sacrifice their own efficiency for the betterment of cellular organization. This inverse performance between two levels can only occur in the presence of conflicting objectives, and the conflicting units will impose immense selection pressure on their regulatory mechanism.

## Cellular level properties

The cellular features reflect on the intracellular goals and objectives that are manifested at the biological and chemical and physical levels. Adaptability, robustness and efficacy of biological systems govern the process at the biological level, and competition, cooperation and coordination among functional products govern processes at the chemical level. Further, thermodynamics, special and temporal constrains govern processes at the physical level. These three levels which comprise the physical, chemical and biological processes operate concurrently by influencing each other during functional organization.

### Adaptability

**Figure 2:**
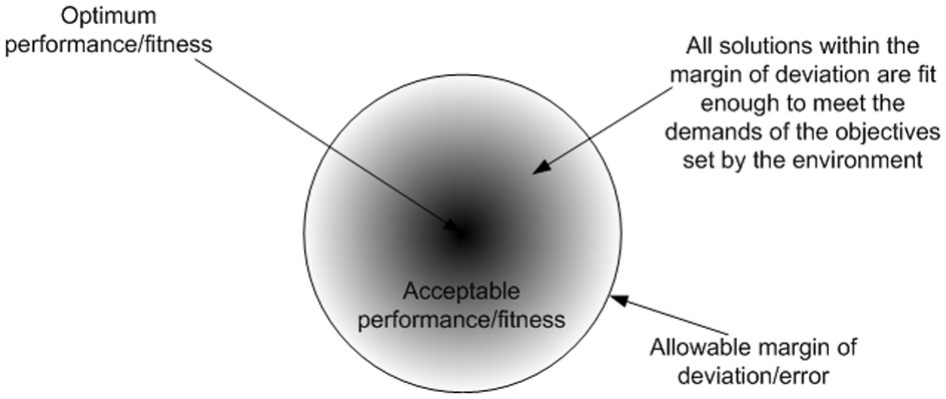
**The outcome of the law of sufficiency is diversity in solutions, where the fittest solutions converge into an attractor basin**. The diversity in outcomes could give rise to the diverse solutions observed in nature. If perfection is the norm, there will be no room for deviation or defects in the outcomes, eventually leading to uniformity in solutions.

Adaptation is a learning process which is associated with Intelligence. The most appropriate definition for intelligence, that covers all computational intelligence approaches, is “the capability of a system to adapt its behavior to meet its goals in a range of environment” (Fogel, 2006). The ability to learn or adapt is one of the hallmarks of intelligent systems. This can also be witnessed in biological cells, where cellular intelligence emerges as an organizational/system level property. The mechanism that drives this intelligent behavior is reinforcement adaptation, which is ubiquitous to biological systems. Reinforcement adaptation is facilitated via a critic, which follows a general principle that serves to guide the adaptive process. These phenomena is observed in cooperative binding (Stefan and Le Novère, 2013) of biomolecules and transcriptional memory (Flintoft, 2007) where the level of response varies which prior stimulus. Biological systems can be assumed to follow the *law of sufficiency*, which states that if a solution is good enough, fast enough, and cheap enough, it is sufficient (Eberhart and Shi, 2007). Hence the suitability of a solution (i.e., fitness) is not an absolute measure, rather it is a relative measure (i.e., how good the solution is relative to other solutions). Figure 2 shows the outcome of the law of sufficiency. The proactive nature of cellular behavior results from the collective organization of biomolecules and their interactions in space and time. Each biomolecule is simply reacting in a determinate way to stimuli and in-turn responding by stimulating other biomolecules to regulate activities amongst them. The uncertainty of when, where and what inter molecular interactions may occur is a stochastic process. However, molecular interactions that lead to meaningful outcome (i.e., either positive or negative for a cell) are relatively deterministic and this is determined by the affinity of the molecular domains to other molecules. For example DNA or protein sequences will determine the potential interactions that can happen in their functional forms. However, when and where these potential interactions may happen is a stochastic process. Various activities are required to provide system wide responses to perturbations. However these activities have limitations, and have to be regulated in terms of when, where and what activities should occur to provide timely responses to perturbations in a constrained environment. As a result, various stages of regulation have evolved in anticipation of perturbations, which facilitate transformation of the activities of functional products, which are merely reactive to a collectively proactive organization. The presence of higher stages of regulation such as translational and post-translational regulation, facilitate the anticipation of recurring perturbations, which also improves the performance of the cell.

**Figure 3:**
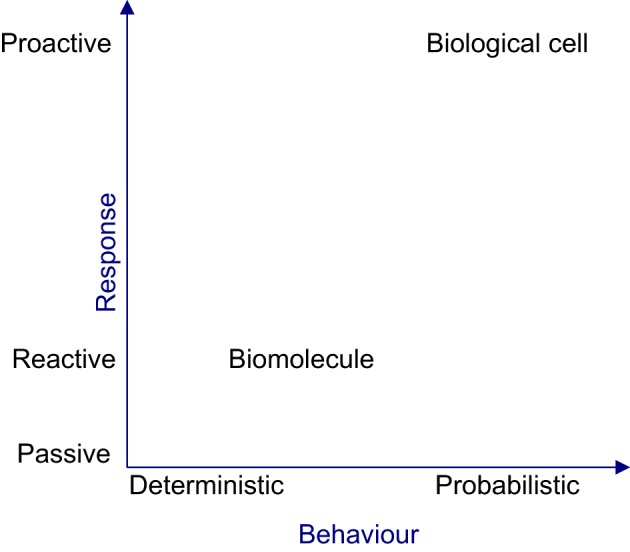
**The nature of biomolecule and the biological cell**. From the reactive and deterministic nature of biomolecular activities, the complex, non-deterministic and proactive cellular behavior will emerge.

From a reductionist perspective, the organizational properties evident at the cellular level such as efficacy, robustness and adaptability, cannot be perceived by characterizing the biomolecules. In the context of reductionism, the cell is perceived tangibly as its constituent biomolecules migrating, physically interacting and causing the density of biomolecular populations to fluctuate in space and time. However, this perception is misleading, since the cell is a collective of autonomous biomolecules exhibiting cohesiveness only at a holistic level. Hence, observing individual biomolecular activities will not provide vital insights about cellular level properties. Moreover the intra-organizational performance of a cell cannot be directly observed or empirically measured, because this requires analysis of the performances of biomolecular species via their activities, analysing the contributions of basic tasks to the complex global tasks of the cell and tracing causalities via causal links amongst biomolecular activities. At an organizational level, cellular behavior can only be probabilistically determined, since causalities occur due to concurrent biomolecular activities. Figure 3 shows the deterministic and reactive nature of biomolecules giving rise to a cellular organization, which is probabilistic and proactive in nature. The deterministic nature of biomolecular behavior can produce coordinated behavior amongst biomolecules, causing reproducible or rhythmic intracellular organizational behavior, in the face of perturbation and uncertainty.

### Robustness

**Figure 4:**
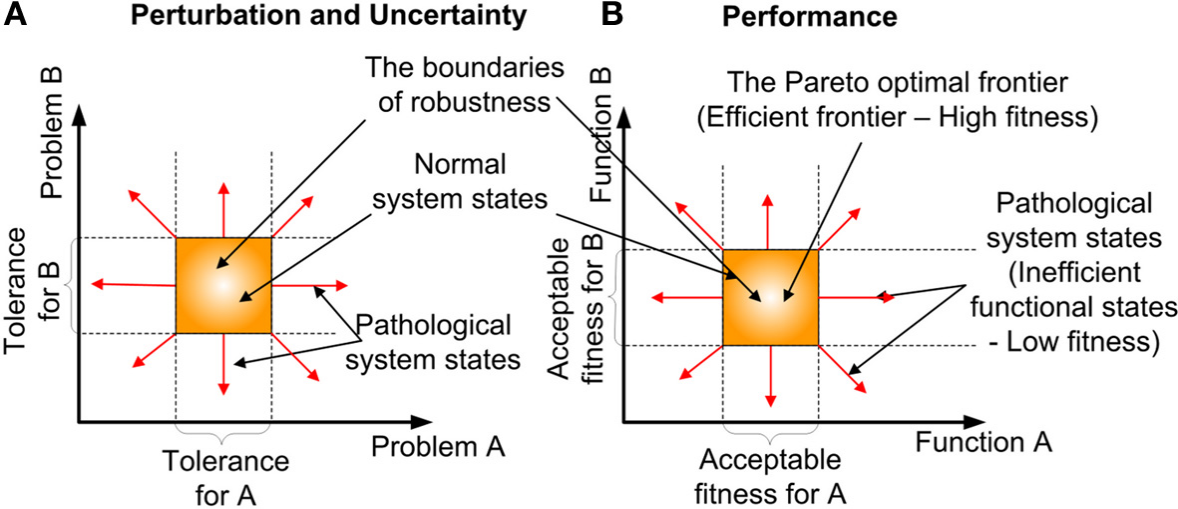
**The formation of robustness and the associated biological equilibrium. (A)** Perturbation and Uncertainty- The existence of the normal system phases (biological equilibrium within the bounds of robustness) boundaries in 2-dimensional problem space. **(B)** Performance—The existence of Pareto optimal frontier (The region of high fitness) boundaries in 2-dimensional function space.

*Robustness* is an organizational/system level property (Kitano, 2004, 2010), which is defined as “the ability to maintain performance in the face of perturbation and uncertainty” (Stelling et al., 2004). The difficulty of comprehending how robustness is accomplished at the cellular or molecular level is due to its intimate link with the complexity of cellular systems (Stelling et al., 2006). However, articles about robustness at these levels have just begun to emerge (Hartman et al., 2001; Felix and Wagner, 2006; Wagner, 2008; Yafremava et al., 2013). An important realization is, that robustness is concerned with preserving the functions of a system rather than system states. This distinguishes robustness from stability or homeostasis (Kitano, 2007). *Homeostasis* is a process, that preserves the state of the system rather than its function. Robustness determines the boundaries (see Figure 4) of the multi-dimensional problem (i.e., perturbation and uncertainty) and the function (i.e., performance) space, in which biological equilibrium can exist (Periyasamy et al., 2009). *Perturbation* defines the extrinsic (environmental) stimulus and intrinsic (programmed) stimulus. *Uncertainty* defines the stochastic nature of the constraints, such as the intervals between biomolecular activities and the availability of resources, which the cell cannot produce. In the context of biological adaptation which spans from physiological to reproductive timescales, function is defined as the progression along some causality, to the goal or successful outcome (Dusenbery, 1992). Some of the factors that contribute to robustness are redundancy and degeneracy, plasticity and concurrency. *Degeneracy* (Edelman and Gally, 2001; Felix and Wagner, 2006) is the ability of different solutions to perform the same function, such as the enzyme's performance can be maintained by altering its processing time or abundance. In contrast *Redundancy* occurs, when the same function is performed by identical solutions. Also redundancy refers to the degree of replica. One of the outcomes of degeneracy is the pleiotropic (Hodgkin, 1998; Lobo, 2008) and polygenic nature of the functional products, where they positively and negatively influence multiple cellular functions, concurrently. The term functional product is currently more favored, than the term gene product, due to changing views of genes (Gerstein et al., 2007). Although degeneracy provides flexibility (many options) for the cell to arrive at a solution (i.e., possibly accelerate adaptation), it adds to the complexity in recognizing the contributions and compensatory adjustments made by different options to the solution. *Plasticity* is the ability of the system to readily adapt to new, different, or changing requirements (Garnier et al., 2007). *Concurrency* manifests in the existence of redundant and specialized biological entities, such as diverse biomolecular species and cell types. The effects of robustness are sensitiveness (fluctuation of performance to perturbations) and adaptability. Robustness facilitates adaptability by accumulating variations whilst maintaining a functional phenotype(Wagner, 2008), such as silent or neutral mutations in the genome.

### Efficacy

Efficacy refers to the effectiveness in responding to the intracellular objectives. The intracellular organization has the ability to efficiently adapt within the bounds of biological equilibrium and gracefully degrade its performance, when functional/performance requirements, perturbation or uncertainty levels demand more than the capacity of robustness. Hence, not only does the biological cell, which is constrained by its genome, maintain performance within the capacity of its robustness, but it also has the ability to reconfigure the responsiveness at the genome level to meet the performance demands of the dynamically changing capacity of robustness. The fitness/effectiveness of solution in the internal cellular organization is constantly being evaluated and it is the measure of performance with respect to an objective. That is how well an intended task is being fulfilled. Although every functional product has a purpose (intended activity), ultimately their contribution to the overall performance of the cellular organization, which in turn contributes to the reproductive success, is essential to understanding of their impact from the bottom up. These functional products will have positive contributions to sustaining biological equilibrium, when their activities are performed when required. However, when their activities are silenced or performed when not required, it can have a negative contribution to sustaining biological equilibrium. This is due to the fact that biomolecular activities are directional/vectorial in terms of their causality (cause and effect), which contributes to the transformation of the cellular organization's equilibrium state, either toward or away from equilibrium, depending on the phase of the cell. Hence the purpose of the functional product in the context of its higher organization (cell) depends on the circumstance, in which the activities are performed. In a normal system, various feedback mechanisms formed by regulatory switches, which span from transcriptional to post translational level, ensure the activities occur in appropriate circumstance to ensure effectiveness of cellular functions.

## Constraints within cells

The distinction between objectives and constraints in biological cells is that when the constraints are subjected to selection pressure they eventually become one of the focuses of the system and will transform into a goal/objective which will have to be pursued by the system for its persistence. Biological cells mostly pursue objectives which are temporal in nature and strive to explore and exploit solutions with respect to time within the bounds of the spatial and thermodynamic constraints. Further, uncertainty (Pearson, 2008; Johnston, 2012; Viney and Reece, 2013) adds to the constraints in meeting the objectives of the cell.

### Uncertainty within cells

The process of biological adaptation which spans from physiological to reproductive timescales involves self-organization and selection, which contributes to the optimization of biological systems. These two mechanisms, which are facilitated by feedback and reinforcement mechanisms, should occur with acceptable fidelity to ensure persistent behavior in biological systems. The cell's ability to organize implies, that it has the ability to optimize cellular activities under various perturbations and uncertainty. The existence of uncertainty in the cellular environment, for which the genome has no control, is due to the presence of faulty activities, unpredictability of causal activities inherent due to concurrency, and the downstream amplification of activities. For example, during the course of evolution an error frequency of about 10^−4^ per amino acid residue, has been selected to produce the greatest number of functional proteins in the shortest time (Stryer, 1988). The ability to organize depends on the predictability of biomolecular activates, which have to significantly dominate uncertain activities. Due to the uncertain nature of the cellular environment, cellular physiology is driven by the most probable molecular activities that occur, based on the constraints in their local environment. Constraints reduce uncertainty by guiding the system. The main constraints for molecular activities include the cost of the activity in terms of time and energy (i.e., enzyme turnover cycle), the spacetime interval amongst the activities (i.e., invariant interval between the two activities/events, which takes into account both spatial separation and their temporal separation), and the stability and availability of reactants (biomolecules) to participate in the activity. The uncertainty involved in spacetime intervals amongst activities, depends on the probability at which respective reactants meet. Biomolecules utilize three kinds of diffusion search spaces. These are one dimensional (along DNA), two dimensional (within the membrane) and three dimensional (in the cytosol) to find their counterparts which initiate the activities. However, the cost of biomolecular activities has been the major constraint (limiting factor) in cellular physiology, since the amount of time required for various biomolecular activities, significantly dominates the time requirements for diffusion mediated encounters.

The stability of functional products also plays a major role in the self-organizing process of the cell, because it determines the functional ability of these molecules. The main factors, which affect the stability of molecules, are temperature, pH and vulnerability to destruction (Plotkin, 2011). Proteins are the molecular machines of the cell and they have evolved to be the major contributors to the organizational dynamics of the cell. Proteins exist in various stages of the lifecycle (Belle et al., 2006) and differ noticeably in their half-lives (Bachmair et al., 1986) (see Table 1), which reflects on their stability. While some are destructed very rapidly (typically enzymes), others are very stable (mechanical proteins). In Proteins, the half-life is determined to a large extent by its amino-terminal residue, which acts as a signal for stability and has been retained over the course of evolution. There is a complex interplay between protein degradation, its regulation and other determinants of protein metabolism (Saric and Goldberg, 2006). The cellular organization has adopted this susceptibility of biomolecular degradation as non-specific negative feedbacks, which contribute to the internal organization of the cell.

**Table 1:** **Half-lives of cytosolic proteins which depend on the nature of their amino-terminal residue (Adapted from Stryer (1988)**.

**Amino-terminal residue**	**Half-life**
**STABILIZING**	
Methionine	
Glycine	
Alanine	>20 h
Serine	
Threonine	
Valine	
**DESTABILIZING**	
Isoleucine	~30 min
Glutamate	~30 min
Tyrosin	~30 min
Glutamine	~10 min
Proline	~7 min
**HIGHLY DESTABILIZING**	
Leucine	~3 min
Phenylalanine	~3 min
Aspartate	~3 min
Lysin	
Arginine	~2 min

*While some are destructed very rapidly (typically enzymes), others are very stable (mechanical proteins). In Proteins the half-life is determined to a large extent by its amino-terminal residue, which acts as a signal for stability and has been retained over the course of evolution*.

### The impact of time and energy

**Figure 5:**
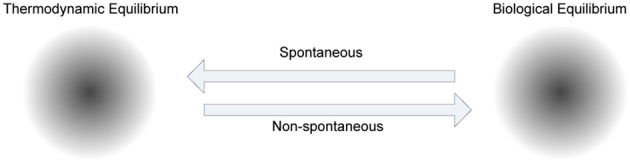
**The role of metabolism in cellular homeostasis**. Various steady states of biological systems, which have emerged to maintain biological equilibrium far from thermodynamic equilibrium, attract non-spontaneous processes to increase order, whereas thermodynamic equilibrium attracts spontaneous processes to decrease order. The trajectory between these two biochemical system phases is controlled by metabolism, where anabolism dominated by non-spontaneous processes, and catabolism dominated by spontaneous processes, are coupled mostly using ATP as a shared medium.

The role of energy in biological adaptation that spans from physiological to reproductive timescales has been emphasized in “thermoeconomics,” as the productivity, efficiency and profitability of various mechanisms for capturing and utilizing available energy to build biomass and do work (Corning, 2002). In metabolism there is a net energy gain in catabolic activities, and a net energy loss in anabolic activities. In biochemical systems, the energy released by catabolism is utilized to drive the synthesis of Adenosine Tri Phosphate (ATP) (known as the currency of energy), which in turn is used for anabolism. Since ATP is released to a common pool and used as a currency the cells have the flexibility to utilize it for any activity that requires it. To facilitate this enzymes play a crucial role in metabolism, because they drive biologically desirable but thermodynamically unfavorable reactions by coupling them to favorable ones. The self-organization processes in cells are non-spontaneous, because energy is required to produce various functional products to maintain order in the cells (see Figure 5).

This specificity has constrained and guided self-organization in biochemical systems. The constant energy flux (energy dissipation) between spontaneous and non-spontaneous processes provides instability, which is required for the self-organization process (Prigogine, 1984; Kauffman, 1993). If the metabolic phase of a biological system reaches thermodynamic equilibrium, it will no longer be considered as a living system. The frequency of reproduction of cells will depend on the amount of energy utilized for reproduction. Energy utilized for other mundane activities of the cell can reduce the frequency of reproduction.

Various regulatory switches have evolved to self-organize the cellular environment. While some switches utilize little or no energy (e.g., binding of signaling molecules), others require chemical modifications using high energy bonds (e.g., chemical modifications mainly by phosphate groups and other groups such as acetyl, methyl and adenyl). Activities of functional products are orchestrated via various regulatory mechanisms which range from transcriptional regulation (genetic level), through post-transcriptional regulation, translational regulation (transcript level) and post-translational regulation (protein level). While transcriptional regulation provides slow and globalized cellular responses, post-translational regulation provides rapid and localized cellular responses. Transcriptional response is the most time and energy consuming process, since genetic information has to be transcribed and mostly translated to produce a functional product. In contrast, post-translational response is the least time and energy consuming process, since the functional product is simply switched between an active and inactive state. Further transcriptional regulating is relatively centralized and stationary, while the remaining regulatory mechanisms are mobile and provide rapid, distributed and localized regulation within the cellular environment. Regulations facilitate in the timing of functional products' activities. Appropriate timing of activities is essential, because its impact depends on the phenotypic state of the cell.

## Multi-level biological organization

A biological cell is organized into an objective/task hierarchy, which contains various cohesive levels. These tasks range from the level of molecular species, where they are atomic and independent of one another, to basic tasks and finally the cellular level, where the tasks become global, mutually dependent and biological. When more than one biomolecular species is involved in the formation of a basic task, mutual dependency will exist amongst the biomolecular species. Hence there is a gradual transition from functions being independent at the molecular level to mutual dependency of functions at the cellular level. The objectives between the levels of the hierarchy are semantically different. The tasks/objectives range from being physical to chemical and biological, when traversing from molecular resolution to cellular resolution. At molecular resolution, the tasks are physical. At the biomolecular species level, a task is represented by its ensemble activity. At the cooperative level where basic functional units emerge, the objectives are involved in completing chemical tasks. However, at the cellular level objectives have characteristics that are fundamental to living systems. That is efficient use of energy, timely responses to perturbation, persistence and other biological characteristics. Further these system level tasks/objectives are not communicated directly to the constituent biomolecules, rather they are self-maintained in a concurrent manner. Nature is inherently concurrent (Corrado, 2009) and biological systems are no exceptions. Since cellular functions are not maintained centrally, cells have adopted a unique strategy to continuously realize their objectives by eliminating obsolete information from their organization. The propensity of biomolecular degradation by means of random or regulated processes and collective autocatalysis provides an ideal reinforcement adaptive mechanism for a cell. The process of biomolecular degradation can eliminate obsolete biomolecular activities and so keep cellular activities up to date, and recycle resources to maintain cellular activities in a resource constrained and dynamic environment. These mechanisms are ubiquitous cellular processes and are pivotal for intracellular adaptive dynamics (Periyasamy et al., 2008).

Cellular level functions are constrained by lower level functions, many of which are in conflict, so various regulatory mechanisms facilitate in managing these conflicts. The higher level tasks enforce adaptive requirements for lower level tasks. Measuring the performances of the functions within the hierarchy would facilitate understanding of the functional organization of the cell. Multi-objective topology provides a concurrent and hierarchical view of intracellular dynamics. A typical multi-objective optimization scenario will generate a set of dominant solutions, which forms the Pareto optimal frontier (the efficient frontier) (Coello Coello, 2006). Optimization uses a controlled trial and error process, where the cellular system is steered along a path of increasing organization. Pareto optimality is an economic concept, which can be used to study the efficacy of biological cells and distribution of biomolecular activities (Ng, 2004). A Pareto efficient frontier is one, in which any change to enhance the performance of an objective is impossible without making the performance of another objective inferior. This is often the case, when there are conflicts among mutually dependent objectives. A mathematically oriented (quantitative) definition for self-organized behavior has been articulated as (Fleischer, 2005) *“Self-organized behavior in a complex system involving multiple performance measures is a sequence of system states corresponding to movement along a Pareto optimal frontier.”* This defines the best global solution that can emerge based within the constraints.

For example, aerobic and anaerobic respirations are dynamic solutions, which have emerged to fulfill the task of liberating energy in the presence and absence of oxygen, respectively. In presence of oxygen, biomolecular activities pertaining to aerobic respiration will dominate, and in absence of oxygen, biomolecular activities pertaining to anaerobic respiration tend to dominate. Hence, these two solutions, although they appear redundant with respect to a cellular objective of releasing energy, are really complementary (i.e., degenerate) with respect to the problem of oxygen content (Rosenfeld, 2002). These adaptive strategies, which are the result of collaborative efforts of biomolecules, provide complimentary solutions for cells. The adaptive mechanisms of biological systems are destined to select appropriate anatomical or physiological solutions (Regenmortel, 2004).

Multi-level interactions deal with associating molecular level activities to cellular level processes. These include representing spatial, temporal and energy constraints, and analysing efficiency, robustness and adaptability from molecular resolution to cellular resolution. Biomolecular activities differ in timescales, which can range from microseconds, as observed in some of the most efficient enzymes, to minutes as observed in transcription and translation of functional products. Although these differences may not appear significant superficially, they have a significant impact on the self-organization of cellular processes.

## Conclusions and prospects

This paper has defined adaptive dynamics of biological cells by utilizing the multi-objective topology which deviates from the conventional network topology based description of intracellular dynamics. Further, it has exemplified biological complexity from molecules to cell by deciphering the functional organization of biological cells via multi-objective representation of the intracellular adaptive dynamics. The paper has characterized the crucial factors involved in biological adaptation occurring in physiological time scales such as adaptability, robustness and efficacy in the context of multi-objective topology which provides a hierarchical and concurrent view of the intracellular dynamics. An appropriate systems biology approach will have to be adopted to model self-ordering and self-organization of biomolecular activities in order to study the emergence of intracellular functional organization. One of the challenges is that the organizational behavior of the cell is not something that can be directly observed or empirically measured. Instead it needs a group of actors to represent the functional products, represent a set of cellular resources utilized by these functional products, capture the results of functional products' activities and a method to evaluate these results (Schut, 2007). This approach provides a novel paradigm which may be harnessed in the development of improved *in silico* representations of cells. The major generic challenge lying at the interface of biology and informatics is that of generating a computational representation of a cell. We propose that these models should represent the self-organizing nature of competing concurrent processes organized into hierarchy; from those activities performed by each biomolecular species, to the tasks delivered by gene families contribute to overall cellular behavior. Models thus developed should enable simulation of functional changes driven by pathological or environmental changes derived from the re-organization of the underlying biological components. Thus, this represents a paradigm shift to promote a bottom–up approach to defining life allowing knowledge generated through reductionism to inform complex biological systems.

### Conflict of interest statement

The authors declare that the research was conducted in the absence of any commercial or financial relationships that could be construed as a potential conflict of interest.
